# Supervised Domain Adaptation for Automated Semantic Segmentation of the Atrial Cavity

**DOI:** 10.3390/e23070898

**Published:** 2021-07-14

**Authors:** Marta Saiz-Vivó, Adrián Colomer, Carles Fonfría, Luis Martí-Bonmatí, Valery Naranjo

**Affiliations:** 1 Instituto de Investigación e Innovación en Bioingeniería, Universitat Politècnica de València, 46022 Valencia, Spain; marsaivi@etsii.upv.es (M.S.-V.); vnaranjo@i3b.upv.es (V.N.); 2 Radiology Department, La Fe University and Polytechnic Hospital, 46026 Valencia, Spain; fonfria_car@gva.es (C.F.); marti_lui@gva.es (L.M.-B.); 3 Biomedical Imaging Research Group (GIBI230-PREBI), La Fe Health Research Institute, 46026 Valencia, Spain

**Keywords:** supervised domain adaptation, MRI sequences, atrial geometry, semantic segmentation

## Abstract

Atrial fibrillation (AF) is the most common cardiac arrhythmia. At present, cardiac ablation is the main treatment procedure for AF. To guide and plan this procedure, it is essential for clinicians to obtain patient-specific 3D geometrical models of the atria. For this, there is an interest in automatic image segmentation algorithms, such as deep learning (DL) methods, as opposed to manual segmentation, an error-prone and time-consuming method. However, to optimize DL algorithms, many annotated examples are required, increasing acquisition costs. The aim of this work is to develop automatic and high-performance computational models for left and right atrium (LA and RA) segmentation from a few labelled MRI volumetric images with a 3D Dual U-Net algorithm. For this, a supervised domain adaptation (SDA) method is introduced to infer knowledge from late gadolinium enhanced (LGE) MRI volumetric training samples (80 LA annotated samples) to a network trained with balanced steady-state free precession (bSSFP) MR images of limited number of annotations (19 RA and LA annotated samples). The resulting knowledge-transferred model SDA outperformed the same network trained from scratch in both RA (Dice equals 0.9160) and LA (Dice equals 0.8813) segmentation tasks.

## 1. Introduction

Atrial fibrillation (AF) is the most common sustained cardiac arrythmia. It has been estimated that 6–12 million people will suffer this condition in the US by 2050 and 17.9 million people in Europe by 2060. Furthermore, this pathology is highly associated with morbidity and mortality factors such as heart failure, ischemic and hemorrhagic strokes and provokes important economic burden [1]. As in-depth knowledge of the physiopathology of the disease remains lacking, the treatment of AF is a complex area.

At this moment, the most common method for treating AF patients is catheter ablation to produce scars and electrically isolate the pulmonary veins (PV) [2]. The success rate of this procedure is significantly higher in patients with paroxysmal AF than in patients with persistent AF which develops fibrotic tissue areas that contribute to the maintenance of AF. To improve the success rate in these patients, clinicians attempt to eliminate the AF driver’s areas guided by complex navigating systems such as Carto or Navex [3].

Accurate segmentation of LA is highly desirable for patient specific scar characterization to select the most appropriate ablation strategy. Furthermore, to plan or guide the procedure in patients with persistent or paroxysmal AF, patient specific 3D electro-anatomical models of both the left and right atrium (LA and RA) are required. These models are obtained by integrating the electro-anatomical information provided by the navigating systems with a 3D geometrical reconstruction of the atria [4].

Notably, 3D patient-specific reconstructions are obtained from the segmentation of MRI and CT images. Cardiac MRI is considered the gold standard for cardiac chamber evaluation including LA and RA. Different image modalities include the balanced steady-state free precession (bSSFP) sequences and late gadolinium enhanced MRI (LGE-MRI). In clinical practice, bSSFP sequences are widely used for LA size characterization due to their excellent blood to myocardium contrast and high spatial resolution [5]. On the other hand, LGE-MRI techniques provide enhanced brightness in fibrotic or infarcted regions, and they are currently used to study the extent of fibrotic tissue in the atria [6]. Furthermore, recent LGE-MRI studies of the atria have led to important advances in understanding and reversing AF [7,8].

**Figure 1:**
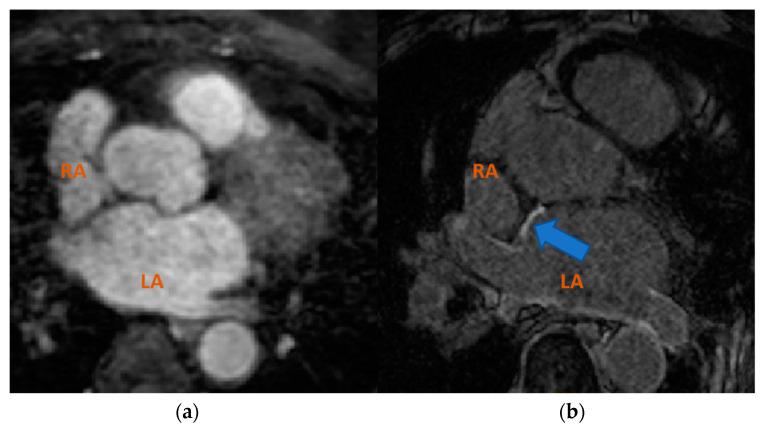
Axial view of different MRI sequences. (**a**) bSSFP and (**b**) LGE-MRI. The blue arrow points to the enhanced brightness of infarcted regions obtained in LGE-MRI. Atrial cavities are labelled as LA (left atrium) and RA (right atrium).

In clinical practice, cardiac MRI segmentation is performed manually by experts which is a time-consuming, labor-intensive and error-prone method. Therefore, there is a need for computational methods for automatic image segmentation. However, high segmentation accuracy remains a challenging task, especially for LA due to its complex geometry [9]. Furthermore, given the diversity of MRI sequences used in the clinical environment, the heterogeneous domain shift is a hot-topic research field [10]. For example, as shown in Figure 1, cardiac regions appear significantly different visually in images acquired using bSSFP and LGE-MRI cardiac acquisitions.

Deep learning (DL) techniques have recently gained popularity in atrial segmentation applications. However, the need for model re-training each time that an external database is provided supposes a significant limitation for their adoption in the clinical practice. Furthermore, it must be remarked upon that although most works focus on the LA segmentation task [11,12,13] due to its complex geometry and PV ablation strategies, the segmentation and reconstruction of the RA has an equally important role for the generation of electro-anatomical models that help to guide and plan ablation procedures, especially in patients with persistent AF [4]. Therefore, there is a growing importance in obtaining models that accurately segment both atrial cavities.

In this work, we present a novel supervised domain adaptation (SDA) framework for LA and RA segmentation tasks. Our approach transfers the knowledge acquired from an LGE-MRI source domain, in which a large, labelled database is available to a b-SSFP-MRI target domain characterized by just a few labelled samples.

The aim is to introduce feature transfer learning techniques in a two-stage 3D U-Net [14] to obtain high accuracy 3D MRI segmentation models capable of segmenting the LA and RA in a target domain composed of few labelled examples. To accomplish this, the supervised domain adaptation technique is formulated in two different scenarios: “different domain, same task” and “different domain, different task” occurrences. Note that this paper extends the preliminary results obtained in [15]. Compared to [15], in this work, we formally formulate the proposed SDA framework, and we extend the validation of our methodology, including an external bSSFP composed of 19 patients with RA annotations. These annotations were manually carried out by the radiologists involved in this work (see Data Availability statement).

The remainder of the paper is organized as follows: In Section 2, we provide an overview of automatic medical image segmentation methods and supervised domain adaptation techniques. In Section 3, the proposed atrial segmentation framework is presented by describing the materials and methodology used. In Section 4, the extensive experiments to validate the proposed approach are described, and results are presented. In Section 5, we provide a discussion of our results, summarize the contributions of this paper and provide some future research directions.

## 2. Related Work

### 2.1. Automatic Medical Image Segmentation

In the field of medical image segmentation, early automated algorithms consisting of thresholding methods and region growing approaches, such as watershed methods, have been widely used. For example, Huang et al. [16] applied watershed algorithms for automatic contouring of breast tumors in US images, and more recently, Masoumi et al. [17] applied watershed algorithms in MRI images with artificial neural networks for liver segmentation. However, the performance of these methods is highly sensitive to boundary contrast and image noise.

By contrast, other region-based methods such as active contours have demonstrated good performance in challenging segmentation problems [18]. For example, Zhao et al. [18] applied active contours with different types of region information for blood vessel segmentation achieving promising results. Ciecholewski et al. [19] used active contours for semi-automatic segmentation of corpus callosum in MR images.

More recently, superpixel-based methods have been proposed to improve the efficiency and robustness of the segmentation procedures. For example, Tian et al. [20] presented a superpixel-based graph-cut model which is iteratively used along with an active contour model to obtain smoother segmentations in prostate MR images. Nguyen et al. [21] applied superpixel methods with multi-atlas segmentation to optimize the image registration process. The above methods are characterized for providing an alternative to machine learning (ML)-based segmentation techniques.

Furthermore, multi-atlas-based segmentation and, more recently, ML algorithms such as support vector machines as statistical classifiers, have also gained interest in the field of cardiac image segmentation and classification. Despite the promising results shown by both the non-ML and ML methods mentioned above, their ad hoc nature and reliability on good initialization have limited their widespread adoption in clinical practice [22].

In recent years, the development of hardware for higher computational power and the growth of clinical databases has enabled the expansion of machine learning (ML) methods into deep learning (DL) algorithms, capable of automatic feature learning and extraction, in the field of image classification and segmentation. Convolutional neural networks (CNNs) with encoder-decoder architectures such as U-Net [23] are widely used in medical image segmentation. Furthermore, 3D extensions of the U-Net [24] have gained popularity as they provide better spatial representation, fully exploiting inter-slice continuity during training [14]. In atrial segmentation tasks, several 3D U-Net variants have been proposed achieving very promising results. The authors of [11] extended 3D U-Net with hierarchical aggregation to obtain better spatial fusion information. The authors of [12] proposed a 3D U-Net with dilated convolutions in the lowest level of the network to extract features spanning a wider spatial range. Furthermore, they cropped the input images around the region of interest as a pre-processing step. For the STACOM 2018 Atrial Segmentation Challenge [25], the authors of [26,27] proposed a 3D U-Net with a dual strategy for localization and segmentation of LA cavity achieving high-accuracy results. More recently, the authors of [13] extended the 3D Dual U-Net strategy by predicting density maps for LA localization with promising results. However, the aforementioned methods [13,26,27] were trained with a large and labelled dataset (the STACOM 2018 challenge dataset), and the authors of [26,27] do not validate their results with an external dataset.

### 2.2. Supervised Domain Adaptation

Given a source domain D_S_ with a corresponding source task T_S_ and a target domain D_T_ with a corresponding task T_T_, transfer learning is the process of improving the target predictive function f_T_(∙) by using the related information from D_S_ and T_S_, where D_S_ ≠ D_T_ or T_S_ ≠ T_T_ [28]. Image domains are expressed with a feature space S and a probability distribution P(x) where X = {x_1_,x_2_, …,x_n_} ∈ S. In this context, domain adaptation (DA) refers to the “different domain, same or related task” scenario where D_S_ ≠ D_T_ due to differences in the samples distribution P(x), for example, when data are acquired from different scanning protocols [29]. The aim of DA is to leverage knowledge learned on a source domain normally consisting of large amounts of data and apply it to a different but related target domain with few labelled data [30]. This work falls in the category of supervised domain adaptation (SDA). Given a source training dataset with pairs D_S_ = {(x^S^_i_, y^S^_i_)}^N^_i=1_ with random variable x^S^_i_ ∈ X^S^ and label y^S^_i_ ∈ Y^S^ and a target training dataset D_T_ = {(x^T^_i_, y^T^_i_)}^M^_i=1_ with x^T^_i_ ∈ X^T^ and label y^T^_i_ ∈ Y^T^, the goal of SDA is to improve f_T_(∙) where M ≪ N and D_S_ ≠ D_T_ [31].

Many DL applications require large amount of labelled data representative of the target task for their optimization. In medical image segmentation, large amounts of labelled target data are often not available or come with high-acquisition and labelling costs. In this case, the typical approach is to use the available datasets (source data) representative of a closely related task but often of different domain due to different scanners or acquisition protocols. SDA techniques were developed to improve the performance of this approach [31].

DA is a widely used technique in transfer-learning scenarios for medical image applications due to its ability to transfer knowledge from different image domains. A comparison of DA approaches for medical image classification and segmentation application can be observed in Table 1. Existing DA approaches include instance transfer and weighting DA strategies where the goal is to align the source and target domains by a transformation of the feature space [32,33,34]. However, these strategies are limited to the “same task” scenario application. With the development of deep-learning methods, feature transfer DA approaches have become increasingly popular due to their ability to transfer information from different domains and different tasks. This is achieved by pre-training a network with a large and available source dataset and fine-tuning the network for a target task [29]. Several works used this approach for image classification tasks [35,36].

More recently, DA approaches have been also applied to medical image segmentation (pixel-wise classification) tasks achieving promising results. The authors of [37] explored unsupervised DA (UDA) with adversarial networks for brain segmentation achieving promising results; however, adversarial training requires subtle parameter tuning. Furthermore, UDA approaches are consistently outperformed with SDA methods which take advantage of a few labelled target samples. Existing works focusing on feature transfer-SDA for medical image segmentation are limited and mostly focus on brain segmentation. For example, Reference [38] explores SDA methods for sub-cortical brain structure segmentation by comparing pre-trained networks fine-tuned with small target samples with fully trained networks and achieving similar accuracy results. Reference [39] compares the performance for brain lesion segmentation in a domain-adapted network and a network trained with the same examples “from scratch” achieving significantly higher accuracy results in the domain adapted network. In the field of cardiac MRI segmentation, the authors of [10] pre-trained a network with bSSFP MR images and fine-tuned the resulting models for LGE-MRI target domain with a few target samples for ventricular segmentation task outperforming the same network without DA. However, in this work, the multi-class segmentation task is kept the same across the different domain instances. As can be observed in Table 1, previous segmentation works focusing on feature transfer with network pre-training have limited their application to same source and target task scenario.

To the best of the authors’ knowledge, no previous works have explored the field of SDA for both LA (T_S_) and RA (T_T_) atrial segmentation tasks. Inspired by this, our purpose here is to formulate and give a solution to the two-fold SDA scenarios, i.e., D_S_ ≠ D_T_ and T_S_ = T_T_ and D_S_ = D_T_ and T_S_ ≠ T_T_, improving the ability of generalization of atrial segmentation models making steps forward in the adoption of computer-aided diagnosis systems based on deep learning in the clinical practice.

## 3. Materials and Methodology

### 3.1. Materials

Three public databases coming from different sources were used to develop and evaluate the proposed SDA framework for LA and RA segmentation.

Database 1 was a result of the STACOM 2018 Atrial Segmentation Challenge. It contains 100 3D LGE-MRIs images from patients with AF and their corresponding LA binary segmentations. The data resolution is 0.625 × 0.625 × 0.625 mm^3^. All volumes are characterized by the same number of slices (88), and there is little variation in terms of image contrast among samples [25].

Database 2 was publicly released by the Multi-Modality Whole Heart Segmentation (MM-WHS) challenge, in conjunction with MICCAI 2017 [40]. It contains 20 volumetric MRI images from human patients with a data resolution of 0.8–1 × 0.81 × 1–1.6 mm^3^ and the corresponding binary segmentation masks of both RA and LA. All volumes had between 256 and 512 slices each. In this work 19 volumetric images were used due to quality controls. This dataset was acquired with a navigator-gated 3D b-SSFP sequence; therefore, it is considered as a dataset of different domain to Database 1 [41].

Database 3 was provided by STACOM 2013 Left Atrial Segmentation Challenge in collaboration with MICCAI13 [42]. It contains 30 volumetric MRI images also acquired using 3D bSSFP sequence with a voxel resolution of 1.25 × 1.25 × 2.7 mm^3^ and the corresponding ground-truth binary segmentation of the LA cavity. All volumes had between 300 and 400 slices each. This dataset contains a variety of quality levels in the following proportions: 19 high and moderate quality and 11 with local artefacts and high noise [43]. In this work, a quality control protocol was applied selecting the 19 patients with good and moderate quality.

In addition, to further extend the validation of the RA segmentation models, a RA ground-truth was generated from this database. The RA ground-truth was obtained by manual segmentation performed by a radiologist (CF) (see Data Availability statement).

### 3.2. Methodology

#### 3.2.1. Domain Adaptation

The scarcity of large and pixel-wise labelled datasets is one of the main problems of deep-learning applications in the field of medical image segmentation. Domain adaptation paradigm enables the transfer of knowledge from a large and labelled source dataset to a target dataset of few examples and different domain [29]. In this work, an SDA technique is formulated for two different scenarios. On the one hand, the segmentation of LA cavity in the sample-constrained bSSFP domain (i.e., Database 2) is faced by transferring the knowledge acquired on the 100 labelled LA instances from the source LGE-MRI domain (i.e., Database 1) under a *“different domain, same task”* premise. On the other hand, a *“different domain, different task”* scheme is proposed taking profit from the knowledge encoded during the LA segmentation task on the source LGE-MRI domain (i.e., Database 1) and transferring it to solve a RA segmentation task in a different bSSFP target domain (i.e., Database 2).

Let θ^LGE^_LA_ be a source model associated with D_S_ ≡ D_LGE_ = {S^S^, P(X^S^)} as LGE-MRI image domain with feature space S^S^ and probability distribution of P(X^S^) where X^S^ = {x^S^_1_, x^S^_2_, …, x^S^_n_} ∈ S^S^, T_S_ a LA segmentation learning task and Y^S^ label space of binary LA segmentation masks. In supervised learning, θ^LGE^_LA_ can be optimized by using a pair of samples {x^S^_i_, y^S^_i_}^N^_i=1_ where x^S^_i_ ∈ X^S^ and y^S^_i_ ∈ Y^S^. After the learning process, the model can be used for solving the T_S_ task in D_LGE_ domain.

Inspired by the domain adaptation paradigm, we can define two target models, each responsible of solving different associated learning tasks (T_T_), under D_T_ ≡ D_bSSFP_ = {S^T^, P(X^T^)} image domain and a small label space Y^T^ with both RA and LA segmentation masks available. One model is tasked with LA segmentation (ψ^bSSFP^_LA_) and the other with RA segmentation (ψ^bSSFP^_RA_). The idea is to transfer the learned weights from θ^LGE^_LA_ to improve the predictive function of θ^bSSFP^_LA_ and θ^bSSFP^_RA_ making use of the {x^T^_i_, y^T^_i_}^M^_i=1_ set of samples, where M ≪ N. Note that in the first case we have defined an SDA scenario in which D_S_≠D_T_ and T_S_ = T_T_, whilst in the second, D_S_ ≠ D_T_ and T_S_ ≠ T_T._

In this work, the proposed SDA framework was built upon the dual 3D U-Net proposed by [14] (see Section 3.2.2 for more details). First, this encoder–decoder CNN was pre-trained on the large, labelled source LGE-MRI dataset giving place to the θ^LGE^_LA_ model.

**Figure 2:**
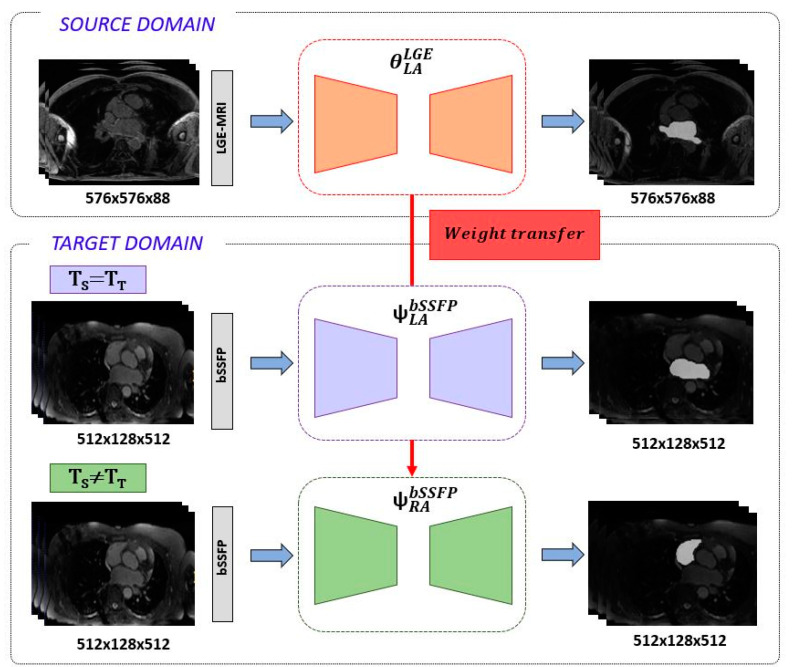
Overview of feature transfer framework. The 3D U-Net is first trained under the LGE-MRI source domain for exclusively LA segmentation. In a second stage, the network is retrained and fine-tuned on the bSSFP target domain for LA and RA segmentation tasks. The red arrow refers to the weight transfer from source model to target model for LA and RA segmentation tasks.

The knowledge acquired on the source domain during the learning procedure was transferred, in a second training stage, to the target domain via weight initialization. In particular, the target encoder, bottleneck and decoder of the dual 3D U-Net were initialized to the resulting θ^LGE^_LA_ weights. Then, the network was fine-tuned using the target bSSFP dataset of different domain and few labelled samples for both LA and RA tasks individually. The proposed SDA pipeline is illustrated in Figure 2, where the final bSSFP adapted models are denoted as ψ^bSSFP^_LA_ and ψ^bSSFP^_RA_ for LA and RA segmentation tasks, respectively. Note that for both segmentation tasks, the loss function, data augmentation scheme and hyperparameters were kept the same.

#### 3.2.2. Dual 3D U-Net

**Figure 3:**
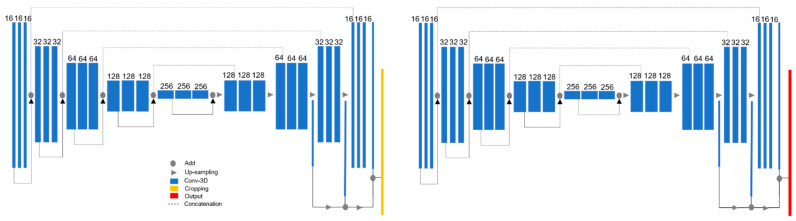
3D Dual U-Net structure proposed by [14]. Blue blocks represent 3D features; orange refers to the cropping interface to crop the region of interest of the first U-Net prediction. Image adapted from [14].

As we mentioned in Section 3.2.1, the proposed SDA framework is built upon the dual 3D U-Net architecture proposed by [14] and illustrated in Figure 3. The 3D U-Net is a specialization of the net proposed by [24] and the implementation follows the work of [44]. It consists of a two-stage network. In the first stage, the network is tasked with locating the region of interest (in this work LA/RA). This preliminary segmentation output is then passed through a processing function that computes the spatial location of the atria and crops the image and masks with a cuboid centered around the region of interest. In the second stage, the training is performed with the cropped images at full resolution. This two-stage approach removes background noise and enables for a more precise segmentation.

The 3D U-Net is a five-level depth encoder–decoder architecture. Each level in the encoder consists of a context module. Each context module is a pre-activation residual block with two 3 × 3 × 3 convolutional layers with a dropout layer (*p* = 0.3) in between. Context modules are connected by 3 × 3 × 3 convolutions with a stride = 2 to reduce the dimensions of the feature maps. In the decoder the low-resolution feature maps are up-sampled by repeating the feature voxels twice in the spatial dimension followed by a 3 × 3 × 3 convolution that halves the number of feature maps. To reduce stochasticity induced by small batch size, instance normalization layers are used instead of the more traditional batch normalization. Skip connections propagate same level features from encoder to decoder. In the decoder, segmentation layers at different levels are integrated via concatenation to reduce the coarseness of the final segmentation output. The activation function used throughout the network is the leaky ReLU with negative slope of 0.01 [44].

## 4. Results

### 4.1. Experimental Configuration

The network was trained several times with fixed experimental settings. The input of the network consisted of volumetric images resized to (224, 144, and 96) with a batch size equal to 1. The number of base filters is 16, and at each level (0–4), the number of filters is computed by (2^level^) × 16.

Training was performed using the Adam optimizer with an initial learning rate equal to 5 × 10^−4^, which reduces by half after 10 epochs if the validation loss is not improving. The convergence is defined as not improving after 50 epochs, and the maximum number of epochs is 500 with 200 steps per epoch. The training–validation split ratio equals 0.8. In addition, data augmentation techniques were used during training to reduce overfitting. Geometric transformations such as image rotations (10 degrees of rotation range), image shifts (in the range of 0.08) and zoom operations (with range equal to 0.2) were applied. Furthermore, to avoid the issue of class imbalance a modified version the Dice loss function introduced by [45] was used for training and validation. The binary class Dice coefficient loss function is defined as follows:

(1) $$L_{DC}=-\frac{2\sum_{i}^{N}p_{i}g_{i}}{\sum_{i}^{N}p_{i}+\sum_{i}^{N}g_{i}+\sigma }$$

where i refers to each voxel of the predicted ($p_{i}$) and ground-truth ($g_{i}$) binary segmentation volumes of size N. The smoothness factor σ equals 0.00001 and was established to avoid the division by 0.

The proposed framework was developed using TensorFlow and Keras, open-source DL libraries for Python, and it was trained on a NVIDIA A100 Tensor with 40 GB RAM. The training time of the network trained with Database 1 (100 LGE-MRI volumes) was 24 h, whilst the network trained with Database 2 (19 bSSFP volumes) was comparatively faster, with the learning process taking 6 h. The evaluation of the predicted segmentations was performed via Dice coefficient and the average surface distance (ASD). The Dice coefficient measures the degree of overlap between the prediction and ground-truth segmentations. The ASD is defined as the average of the minimum distances (voxel-wise) between the ground-truth and prediction object boundaries. The ASD is evaluated using the shortest Euclidean distance of a voxel v to a point P, defined as:

(2) $$\bar{d}\left(v,P\right)={min}_{p\in P}||v-p||$$

### 4.2. Source Domain Model

**Figure 4:**
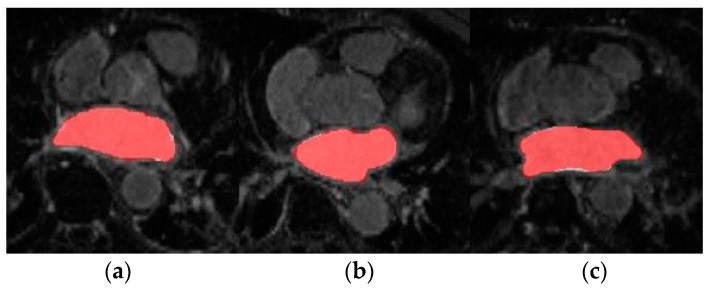
Axial slices of LA segmentations for three representative patients (**a**–**c**) obtained through the LGE-MRI source model. Ground-truth mask in white, prediction mask in red and intersection in pink.

Firstly, the dual 3D U-Net was trained and tested with a large number of labelled images from LGE-MRI (source) domain. For this, Database 1 was employed which contains 100 volumes each of them composed of 88 slices. The aim of this experiment was to pre-train the network and obtain a high-performance model from which knowledge can be inferred. The mean Dice coefficient and standard deviation obtained during evaluation across the 20 testing volumes were 0.9155 ± 0.0270. The segmentation results for three representative patients and slices of Database 1 can be observed in Figure 4.

### 4.3. Supervised Domain Adaptation Models

In a second stage, the learned knowledge θ^LGE^_LA_ in D_LGE_ was transferred under the proposed SDA framework via weight initialization. In this line, the dual 3D U-Net was adapted for D_bSSFP_ by retraining the network with few labelled samples of bSSFP. For this purpose, Database 2 was employed which consists of 19 bSSFP volumetric samples. The SDA was performed twice, once for LA segmentation task and the other for RA segmentation task; thus, two domain-adapted networks were obtained.

Table 2 shows the Dice and ASD metrics of three LA segmentation models, each with a different training strategy and evaluated with bSSFP test samples from Database 2. The training strategies include the source model trained with LGE-MRI images and the models trained with bSSFP samples (Database 2) with and without SDA. This table provides a performance comparison of the domain-adapted network in the D_S_ ≠ D_T_ and T_S_ = T_T_ scenario. We use boldface to highlight the most important results in the tables.

Table 3 shows the Dice and ASD metrics for RA segmentation models with and without SDA approach (D_S_ ≠ D_T_ and T_S_ ≠ T_T_ scenario).

### 4.4. External Database Validation

With the aim of providing an in-depth evaluation of the proposed SDA framework, an external validation was carried out. The idea behind this experiment is to corroborate the ability of generalization of the domain-adapted models. To this end, the trained models for bSSFP domain with and without adaptation were further validated with a testing database of 19 volumetric bSSFP samples with 300–400 slices each (Database 3).

Table 4 and Table 5 show the average inferred Dice coefficient and ASD metric for LA and RA segmentation tasks, respectively. Note that in the case of LA segmentation the source LGE-MRI trained model is included as baseline indicator (Table 3).

To further assess the evaluation performance of each model, qualitative results of the segmentation predictions were obtained.

**Figure 5:**
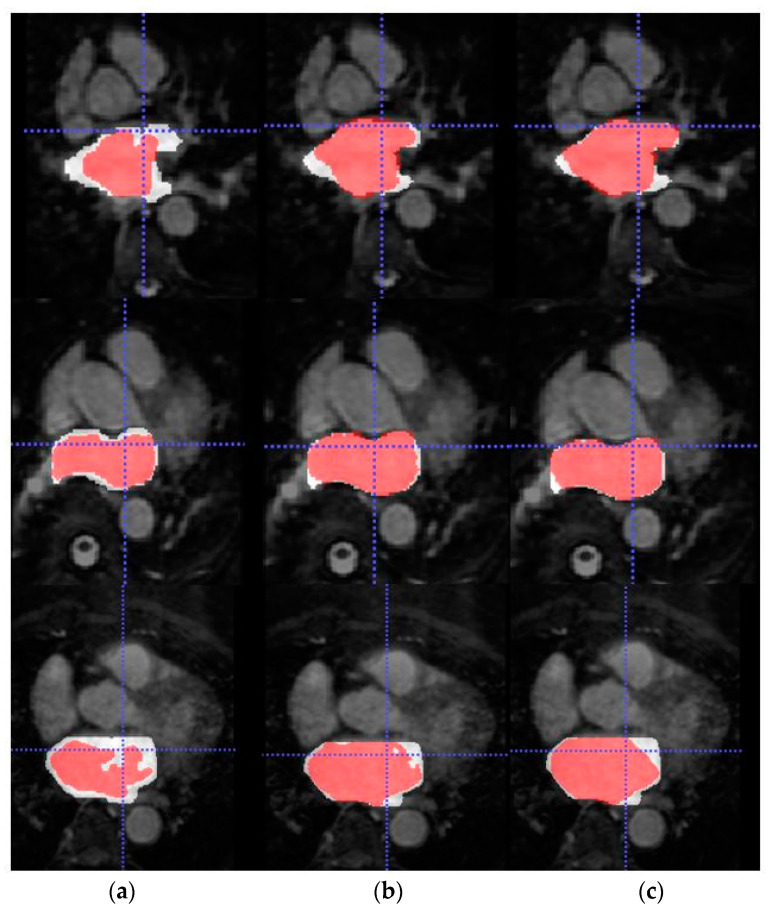
From top to bottom, axial slice of LA segmentations for three patients obtained through models (**a**) LGE-MRI, (**b**) w/o SDA and (**c**) w-SDA. Ground-truth mask in white, prediction mask in red and intersection in pink.

Figure 5 shows the axial view of predictions obtained for three representative patients (and slices) of the testing cohort from Database 3 with the different trained models for LA segmentation.

**Figure 6:**
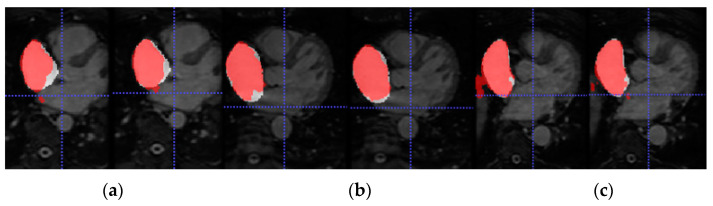
From left to right, axial slice of RA segmentations in networks w/o SDA (left) and w-SDA (right) for 3 patients (**a**–**c**).

Figure 6 shows the axial view of predictions obtained for three representative testing patients (and slices) of Database 3 with the different trained models for RA segmentation.

Furthermore, 3D reconstructions of the atrial cavities were obtained from the testing patients of Database 3 to assess the geometry of the predicted segmentations.

**Figure 7:**
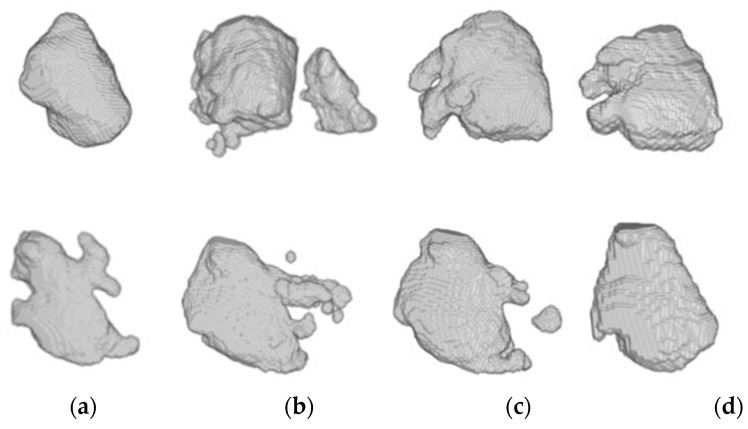
From top to bottom, 3D reconstruction of LA segmentations for two representative patients obtained through the models (**a**) LGE-MRI, (**b**) w/o SDA, (**c**) SDA and (**d**) ground-truth.

Figure 7 illustrates the 3D reconstruction of the LA segmentation obtained by LGE-MRI, bSSFP and bSSFP with SDA-trained models for two representative patients of Database 3 as well as the ground-truth mask reconstruction for comparison.

**Figure 8:**
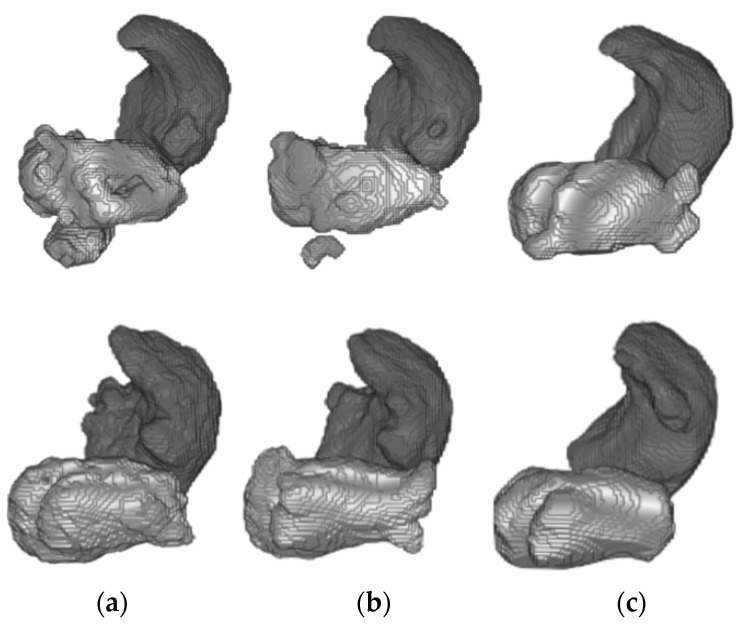
From top to bottom, 3D reconstruction of LA (light gray) and RA (dark gray) segmentations for two representative patients of Database 3 through the models (**a**) w/o SDA, (**b**) SDA and (**c**) ground-truth.

Figure 8 shows the 3D reconstruction of both the LA and RA cavities obtained by the trained models bSSFP with and without domain adaptation for two representative patients from Database 3.

## 5. Discussion

In this section, we review the results obtained in the different experiments presented in Section 4. Firstly, the source network was trained with a large number of annotated LGE-MRI samples (Database 1). As can be observed, the model achieved very high accuracy segmentation results. This is expected as Database 1 corresponds to the data provided by the 2018 LA segmentation challenge [25], and the network proposed by [14] was specifically designed to face this challenge. Therefore, by keeping the same learning parameters, the Dice obtained coincides with the Dice reported in [14] (0.91–0.92). The segmentation masks shown in Figure 5 further highlight the high-accuracy performance of this model.

As we can observe in Table 2 and Table 3, for both LA and RA segmentation the domain adaptation improved the segmentation performance in terms of Dice and ASD metrics. Remarkably, with the proposed SDA methodology the RA segmentation average Dice increased to 91%, and the ASD was reduced to 1.3024 mm. This is particularly interesting as the knowledge was transferred from a source model pre-trained for a different segmentation task (LA), which demonstrates the robustness of SDA approaches applied in a T_S_ ≠ T_T_ scenario. With this approach, models could be trained with a few labelled examples and yield high-accuracy segmentation results regardless of the target segmentation task.

Furthermore, as we can observe in Table 2, the source LGE-MRI model obtained a considerably lower Dice score (from 0.8813 to 0.7773) and higher ASD score (from 1.5460 to 2.9079 mm) when evaluated with Database 2. This illustrates how the performance of a U-Net trained from a particular source domain, when transferred to a different target domain, can drop unexpectedly, otherwise known as the domain shift problem. Compared to the Dice average of evaluated methods presented in the Multi-Modality Whole Heart Segmentation Challenge with the same database (0.815 for LA segmentation and 0.831 for RA segmentation) [41], the Dice obtained with our proposed method for both LA and RA segmentation is considerably higher; note that in the RA segmentation task, the Dice increment is almost 10%, which suggests that supervised DA and transfer learning is an effective method for obtaining good quality LA and RA segmentation. Although each testing volume contains between 100 and 200 image slices, to obtain more representative results, an increased number of testing patients are needed. For this reason, the proposed SDA framework is also assessed using an external database.

Table 4 and Table 5 summarize the comparison results, in terms of Dice and ASD scores, of the trained models evaluated with an external bSSFP database for LA and RA segmentation, respectively. As we can observe, the SDA models achieved superior segmentation performance for both segmentation tasks, obtaining an average Dice and ASD score of 0.8405 and 2.2574 mm, respectively, for LA segmentation and an average Dice and ASD score of 0.7993 and 2.8816 mm, respectively, for RA segmentation. These results suggest that domain adaptation also improves the generalization capability of segmentation models. The results in Table 4 show that both bSSFP-trained models outperformed the source LGE-MRI trained model, achieving a 10% of gain in Dice accuracy from the LGE-MRI source model to the SDA-bSSFP model. This illustrates the importance of adapting the domain of the models to the target segmentation. Furthermore, it should be noted that the gain in Dice accuracy between bSSFP models with and without SDA is similar in both RA and LA target segmentation tasks (2–3%), further proving the robustness of SDA methods. Regarding the ASD metrics, an increased number of samples in the test set would be needed to properly assess the statistical significance of the results.

Due to the absence of previous works that employ Database 3 solely as an external validation dataset, we are not able to compare the generalization ability of our proposed model. On the other hand, a visual representation of the increased performance of the models with SDA can be seen in Figure 5 and Figure 6 for LA and RA segmentation tasks, respectively. As we can observe, the blood pool prediction segmentation of the LA and RA performs best in the SDA model.

In addition, Figure 7 and Figure 8 depict the 3D reconstruction of the prediction segmentation obtained by each trained model. Specifically, Figure 8 shows the increased performance of LA segmentation from the source model to the SDA bSSFP model as the geometry of the cavity becomes more similar in shape to the ground-truth reconstruction. Furthermore, the domain shift problem can be qualitatively assessed as the LGE-MRI source model clearly fails to generate an atrial geometry anatomically similar to the ground-truth. Figure 7 shows the combined reconstruction of LA and RA predictions in bSSFP models with and without SDA. As we can observe with SDA, the overall geometry of the atrial cavities is closer to the ground-truth.

In light of the results, transfer learning with a supervised DA approach enables that a model trained with a few labelled samples could yield improved segmentation accuracy by successfully leveraging knowledge from different image domains and segmentation tasks. Furthermore, SDA models demonstrate a greater generalization capacity when evaluated with external databases without retraining.

The limitations of this work remain similar to those presented in the previous work [15]. Specifically, more testing image data would be required to further validate the generalization capability of the obtained model and to adequately evaluate the statistical significance of the results. Given that the results promisingly show that the SDA-adapted models show good performance in a bSSFP image domain without the need for retraining, image datasets of more different domains would be needed to test if the improved generalization ability can be extended to other domains. Due to the limited amount of public cardiac datasets with LA and RA annotations, this constraint remains challenging to resolve; nevertheless, since the last presented work [15], a dataset with RA annotations performed manually by a radiologist has been generated as an attempt to reduce this main limitation.

## 6. Conclusions and Future Work

In conclusion, supervised DA approaches enable deep neural networks to be trained with a few labelled examples to improve segmentation accuracy results by leveraging the knowledge of a larger source dataset of a different domain. Furthermore, our proposed framework has demonstrated that leveraging knowledge from different segmentation tasks (LA to RA) could be useful for improving segmentation accuracy.

These results are the first step toward the integration of automatic segmentation algorithms in the clinical practice for AF management where accurate segmentation models of both atrial cavities are needed to ensure the success of AF ablation procedures. Furthermore, the amount of manually labelled samples to train automatic computational models is limited in clinical practice, especially for the RA. Therefore, it is extremely useful to be able to leverage knowledge from models trained on LA segmentation tasks for RA segmentation. In addition, as acquisition protocols vary in different centers, there is a need to take advantage of the available clinical datasets even from a different domain for computation learning. Our proposed method has proven to have greater generalization capacity when trained with an external testing database, thus potentially eliminating the need for retraining for each acquisition protocol employed.

The trained models should be further evaluated with external databases of different domains and a large sample size to explore the role of SDA approach in increasing segmentation accuracy and the generalization ability of the network. Furthermore, it would be interesting to evaluate the obtained SDA models with RA segmentations manually generated by other trained experts to assess the effect of inter-user variability on the results obtained.

As a future line of research and given the promising results obtained by SDA techniques, we propose extending this framework to generate multi-organ segmentation models. Finally, the extent of the differences between source and image domains and source and image tasks for SDA to remain effective should be further investigated (i.e., the effect of introducing noise to the source domain, also known as adversarial attacks).

## Figures and Tables

**Table 1:** Overview of DA applications. UDA: unsupervised domain adaptation, SDA: supervised domain adaptation. Transfer type column refers to DA transfer technique, i.e., feature adaptation, instance transfer (weighting or aligning samples), or feature transfer (pre-training on same or different auxiliary task).

First Author	Application	Task	Transfer Type	Pros/Cons
UDA				
Kamnistas et al. [37]	Segmentation of abnormalities	Same	Feature adaptation	No labels/subtle parameter tuning
SDA				
Conjeti et al. [32]	Tissue classification	Same	Instance transfer, align	Considers local and global domain/only align similar features
Wachinger et al. [34]	AD classification	Same	Instance transfer, weights	Good accuracy with small target labels/weight selection
Menegola et al. [35]	Melanoma classification	Different	Feature transfer, pre-training	Fine-tuned outperforms baseline models/relies on size of source dataset
Ribeiro et al. [36]	Polyp classification	Different	Feature transfer, pre-training	Comparative study/conclusions not always hold
Kushibar et al. [38]	Brain segmentation	Same	Feature transfer, pre-training	Fine-tune with small target sample/only applied to same task
Ghafoorian et al. [39]	Brain lesion segmentation	Same	Feature transfer, pre-training	Last dense layers fine-tuning/only applied to same task
Vesal et al. [10]	Ventricle segmentation	Same	Feature transfer, pre-training	Good results in multi-class segmentation/only applied to same task

**Table 2:** Dice coefficient and ASD for LA segmentation on bSSFP (Database 2) test data set.

Methods	Dice				ASD [mm]			
	Patient 1	Patient 2	Patient 3	Mean ± σ	Patient 1	Patient 2	Patient 3	Mean ± σ
LGE-MRI	0.7155	0.8030	0.7275	0.7487 ± 0.0474	2.3120	1.8907	4.5210	2.9079 ± 1.4128
w/o SDA	0.8693	0.8756	0.8736	0.8729 ± 0.0032	1.3614	1.2137	2.3517	1.6422 ± 0.6188
W-SDA	0.8781	**0.8948**	0.8707	**0.8813 ± 0.0124**	1.2522	**0.9726**	2.4132	**1.5460 ± 0.7639**

**Table 3:** Dice coefficient and ASD for RA segmentation on bSSFP (Database 2) test data set.

Methods	Dice				ASD [mm]			
	Patient 1	Patient 2	Patient 3	Mean ± σ	Patient 1	Patient 2	Patient 3	Mean ± σ
w/o SDA	0.9162	0.8773	0.8892	0.8942 ± 0.0199	0.9683	1.7313	2.1606	1.6201 ± 0.6039
W-SDA	**0.9273**	0.9043	0.9166	**0.9160 ± 0.0115**	**0.7278**	1.5357	1.6438	**1.3024 ± 0.5006**

**Table 4:** Average Dice coefficient and ASD for LA prediction segmentation on bSSFP (Database 3) test data set.

Methods	Dice	ASD [mm]
LGE-MRI	0.7552 ± 0.0640	2.9208± 1.6903
w/o SDA	0.8186 ± 0.0703	2.6311 ± 1.4620
w-SDA	**0.8405** ± 0.0614	**2.2574** ± 1.5047

**Table 5:** Average Dice coefficient and ASD for RA prediction segmentation on bSSFP (Database 3) test data set.

Methods	Dice	ASD [mm]
w/o SDA	0.7724 ± 0.0996	3.2821 ± 1.8413
w-SDA	**0.7993** ± 0.0928	**2.8816** ± 1.6158

## Data Availability

The manual annotations of the RA of dataset [40] generated in this study can be found in https://github.com/marsaivi/SDA-RA-Dataset (accessed on 14 May 2021).
